# Decoding the regulatory trio: The role of Nhd1-OsMYB110-OsMADS15 in rice flowering time

**DOI:** 10.1093/plphys/kiaf323

**Published:** 2025-07-22

**Authors:** Nilesh D Gawande

**Affiliations:** Assistant Features Editor, Plant Physiology, American Society of Plant Biologists; Department of Biological Sciences and Engineering, Indian Institute of Technology Gandhinagar, Palaj 382355, India

Flowering time or heading date is the critical stage in rice that helps plants to acclimatize to diverse seasonal fluctuations. Early-flowering rice cultivars are advantageous because delayed flowering in rice leads to yield losses due to adverse weather conditions compromising photosynthetic organ growth or reducing fertility (Tsuji et al. 2013).

Rice is a facultative short-day plant, where short days promote flowering and long days inhibit flowering. Florigen is a small globular protein encoded by the *Heading date 3a* (*Hd3a*) gene in rice. Rice has 2 florigen genes, *Hd3a* and *RICE FLOWERING LOCUS T1* (*RFT1*), where *Hd3a* acts under short day conditions while *RFT1* acts under long day conditions (Zhou et al. 2021). The florigen activation complex is a group of 3 proteins that promote flowering. The key protein in this complex is one of the two florigens. Day length influences the expression of *Hd3a* and *RFT1* in leaves and sends signals from the leaves to the shoot apical meristem (SAM). Furthermore, Hd3a and RFT1 form a complex with the transcription factor FD and a 14-3-3 protein scaffold, which activate genes responsible for flower development, such as *APETALA1* in *Arabidopsis* and *OsMADS15* in rice (Tsuji 2017).

Nitrate availability creates a U-shaped flowering curve, where an optimal nitrate concentration promotes flowering, while levels above or below this optimal amount delay the process. N-mediated heading date-1 (Nhd1) belongs to the MYB transcription factor family, whose expression begins after the floral transition phase and increases at later floral developmental stages under high nitrogen conditions. Nhd1 regulates flowering through the Hd3a-dependent flowering pathway, which alters the expression of *MADS* genes in the SAM (Zhang et al. 2021). However, *MADS* genes do not have a binding site for Nhd1. Therefore, investigating how Nhd1 regulates flowering and identifying other proteins involved in this process requires further study.

In this issue of *Plant Physiology*, Jin et al. (2025) identified the Nhd1-OsMYB110-OsMADS15 regulatory module, which integrates developmental and nutrient signaling to regulate flowering time in rice (Figure 1). The authors knocked out *MYB110* in rice and found that it significantly delayed flowering in both long- and short-day conditions, whereas plants overexpressing *Nhd1* (*Nhd1-OX*) displayed earlier flowering. The crossing of the *Nhd1-OX* with *myb110* mutants displayed a delayed flowering phenotype like *myb110*, suggesting that *myb110* was epistatic to *Nhd1-OX*. They also found that *MYB110* levels increased significantly in the *Nhd1-OX* plants, indicating that the higher *MYB110* expression may be linked to earlier flowering. These findings indicate that MYB110 has a crucial role in the way that Nhd1 regulates flowering time in rice.

**Figure.**
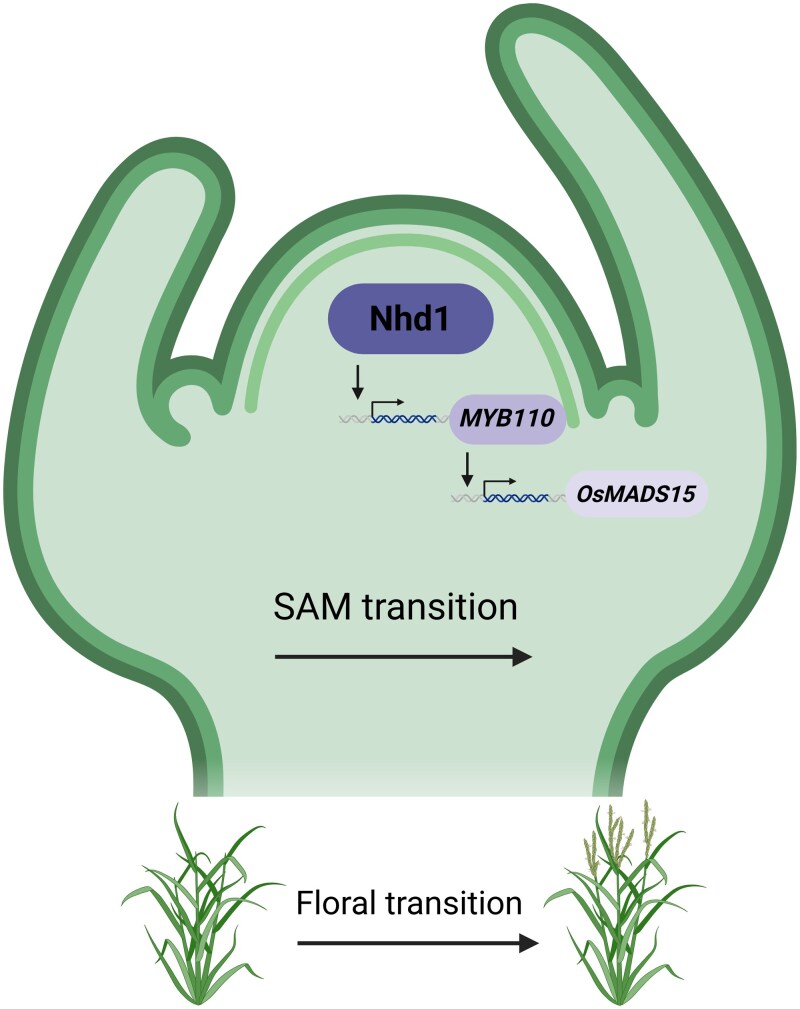
Regulation of flowering time in rice by the Nhd1-OsMYB110-OsMADS15 module. The MYB family transcription factor Nhd1 binds to the promoter of *MYB110*, promoting its expression. Subsequently, *MYB110* activates *OsMADS15*, which is crucial for facilitating the floral transition in the SAM and in the regulation of flowering time. This figure was created with BioRender.com.

Furthermore, the authors used yeast one-hybrid, EMSA, and ChIP-qPCR to show that Nhd1 can bind to the NBS element in the *MYB110* promoter, which likely activates its expression. The *myb110* mutants displayed similar U-curve responses for flowering time to nitrogen supplies as the wild type (WT). This suggests that while MYB110 is important for activation of flowering time by Nhd1, it does not influence how Nhd1 regulates flowering time in response to nitrogen.

The authors investigated whether the delayed flowering time in *myb110* mutants was due to inhibited floral transition. They compared the stem apex phenotypes of *myb110* mutants and WT plants, finding that WT exhibited earlier panicle formation. They discovered that *MYB110* is expressed in the SAM, indicating its potential role in the floral transition. The authors used RNA sequencing to show that *MADS15* expression was significantly inhibited in *myb110* mutants. *MADS15* CRISPR knockout mutants flowered much later than WT, confirming it as a positive regulator of flowering. Overexpressing *MADS15* led to earlier flowering, and plants overexpressing *MADS15* in the *myb110* mutant background flowered earlier than *myb110* mutants but still later than WT plants. The authors used Y1H, EMSA, ChIP-qPCR, and dual-luciferase reporter assay to show that MYB110 directly binds to the Myb binding site (MBS) motif in the *MADS15* promoter. Overall, the findings suggest that MADS15 is one key downstream target of MYB110 that regulates flowering time in the rice SAM.

The functions of MYB110 and MADS15 are crucial for flowering time sensitivity to phosphorus (Pi) supply. WT plants showed delayed flowering under low Pi and accelerated flowering with high Pi. However, *myb110* and *mads15* mutants had significantly delayed flowering regardless of Pi levels, indicating insensitivity to Pi supply. Overexpressing *MADS15* in *myb110* mutants restored the high Pi-induced flowering response. *MADS15* displayed a significant increase under high Pi conditions, while the expression of MYB110 was not significantly induced. Since *Nhd1* mutants can respond to Pi, the threecomponent module does not regulate this response.

In summary, this article demonstrates that Nhd1 activates OsMYB110 to control *OsMADS15* expression and flowering time in rice. Future research could focus on elucidating the specific molecular mechanisms by which the Nhd1-OsMYB110-MADS15 regulatory module interacts with other signaling pathways and environmental factors, particularly in relation to varying nutrient conditions and their impact on flowering time in rice.

## Data Availability

No new data were generated or analysed in support of this.
